# Beyond Accuracy: Methodological Advances for Assessing the Clinical Impact of Infectious Disease Diagnostics

**DOI:** 10.1093/ofid/ofaf489

**Published:** 2025-10-22

**Authors:** Kimberly C Claeys, Andrea M Prinzi, Tristan T Timbrook

**Affiliations:** Department of Pharmacy Science and Health Outcomes Research, University of Maryland School of Pharmacy, Baltimore, Maryland, USA; bioMérieux, Medical Affairs, Salt Lake City, Utah, USA; Department of Pharmacy, Barnes-Jewish Hospital, Saint Louis, Missouri, USA

**Keywords:** diagnostics, hybrid effectiveness-implementation, infectious diseases

## Abstract

Evaluating the clinical impact of *in vitro* diagnostic tests (IVDs) for infectious diseases is complex given their effectiveness depends on context, implementation, and provider behavior. Traditional methodologies for therapy interventions do not adequately capture this complexity, necessitating novel analytical approaches and study designs. This review highlights methodological considerations for improving evidence generation for infectious diseases IVDs. Design and analysis challenges leading to bias and related solutions are reviewed such as the target trial framework. Moreover, novel frameworks such as Benefit–Risk Evaluation of Diagnostics: A Framework, Desirability of Outcome Ranking Management of Antimicrobial Therapy, and Desirability of Outcome Ranking and study designs such as hybrid effectiveness–implementation designs are discussed which allow for holistic ways to assess real-world outcomes. By evaluating IVDs with practical, real-world evidence, tests can better inform clinical decision making, policy, and ultimately patient outcomes.

Assessing the clinical impact of *in vitro* diagnostic tests (IVDs), specifically rapid diagnostic tests (RDTs) for infectious diseases (ID), is essential for demonstrating their value and promoting their adoption in evidence-based practice. Unlike pharmaceuticals, the effectiveness of IVDs depends on how results are used to inform clinical decisions highlighting the need for diagnostic stewardship and implementation considerations throughout a test's lifecycle. In simple terms, traditional study methodologies designed for pharmaceutical interventions to evaluate IVDs will not comprehensively demonstrate their value [1].

The foundation for choosing an IVD depends primarily on the test's specifications, performance, and diagnostic accuracy [2]. However, unlike pharmaceuticals, which receive regulatory clearance based on their impact on clinical outcomes, IVDs are often approved and scaled up for public health use based on accuracy alone. From both a philosophical and practical standpoint, IVDs cannot be fully evaluated until their impact on patient outcomes is understood [3]. Many peripheral components of healthcare delivery affect this understanding, including how the test is implemented (ie, where, how, and with whom it is used), how it is interpreted, and the associated provider behaviors resulting from the test results. To ensure that findings are reliable, relevant, and generalizable, it is crucial to account for as many of these considerations as possible when studying the clinical impact of IVDs. Methodological best practices should be employed to address the complexities associated with measuring the impact of IVDs. Integrating principles of diagnostic excellence (including diagnostic stewardship) and antimicrobial stewardship (AMS) is important, as these principles have been shown to significantly impact care delivery and improve patient outcomes [4, 5].

Whenever possible, implementation science should be leveraged to bridge the gap between evidence and practice, adapting study designs to meet contextual needs and systematically addressing behavior change alongside IVD implementation [6]. Finally, efforts should be made to design studies that address the needs of populations often excluded from primary clinical trials due to methodological limitations and generate evidence that supports test reimbursement, policy changes, and integration into clinical guidelines. This review outlines key methodological considerations for studying the impact of these tests, the importance of diagnostic stewardship and implementation science in clinical research, and the data requirements needed for guideline development, test reimbursement, and enhanced market access.

## METHODOLOGICAL CONSIDERATIONS FOR STUDYING AND DEMONSTRATING THE IMPACT OF DIAGNOSTIC TESTS

**Figure 1.**
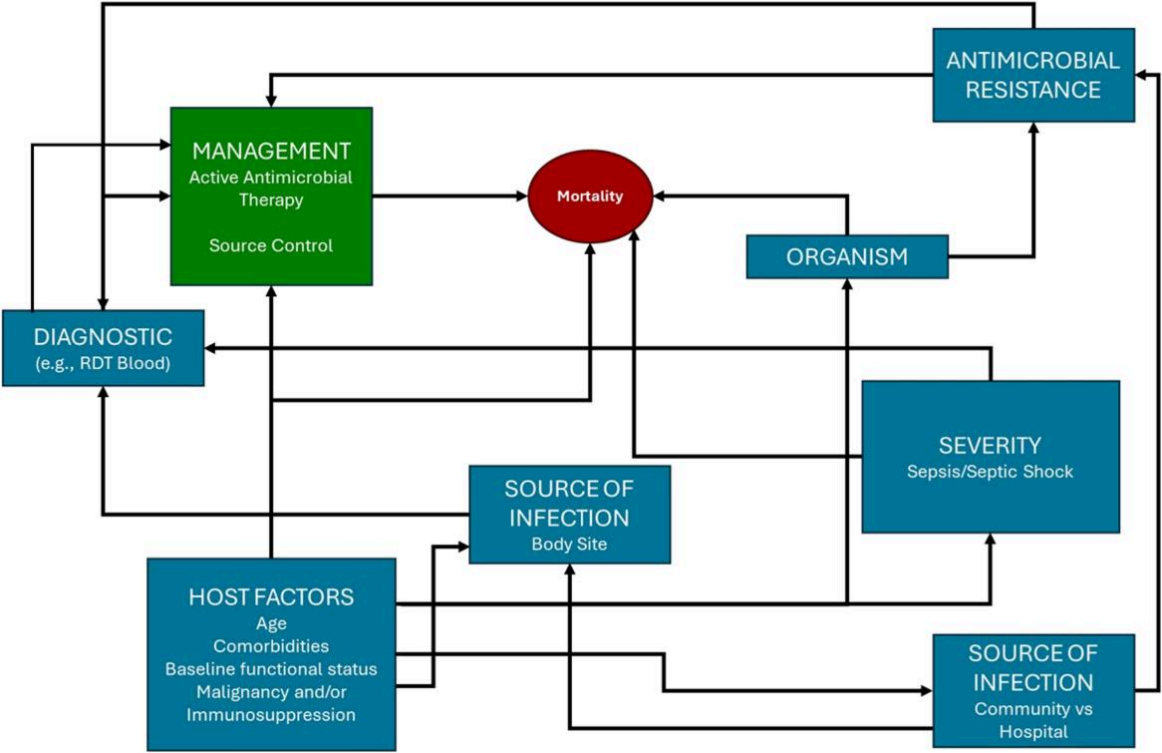
Example of a hypothetical directional acyclic graph (DAG) for *in vitro* diagnostic tests (IVDs) in bloodstream infections.

Studying the impact of IVDs requires careful consideration. Before beginning a study, it is essential to determine whether a clinically relevant opportunity for improvement exists for the primary outcome that the study is designed to assess (Figure 1). This should be established with local baseline quantitative data beyond a subjectively perceived opportunity to improve care (Table 1). Failing to perform such due diligence may lead to undetectable changes due to small baseline event rates and sample size limitations. Moreover, to ensure the capacity of a test for impact, and selection of relevant outcomes, study outcome selection should be driven by the use of existing or explicit development of potential care pathways, including considerations such as resource utilization and patient reported outcomes (eg, values and preferences) [7–9]. Guidance on developing such pathways may be found elsewhere [10]. To ensure clear and specific estimates of effect are established, a study should characterize a specified population, intervention, comparison, outcome, the timing in the care pathway of testing, and the clinical setting (PICOTS) [11]. Finally, in selecting study outcomes, subjective adjudicated primary outcomes or part of a primary composite outcome should generally be avoided given the often very low interrater and intrarater reliability of agreement in such approaches which usually biases results toward the null [12–14].

**Table 1.** Methodological Considerations for IVD Clinical Outcomes Study Design

Step	Key Points	Example
Identify clinically relevant opportunities for improvement	Leverage baseline data to understand population and trends Avoid subjective expert adjudicated primary outcomes where interrater reliability is low Use PICOTS framework	A study assessing the impact of a diagnostic intervention on antibiotic avoidance versus SOC fails to show an impact due to high rate of baseline antibiotic avoidance (ie, in the setting of highly effective antimicrobial stewardship)
Leverage diagnostic stewardship principles in study design	Use knowledge of pretest probability and patient population to determine composition of intervention group	A diagnostic intervention is only randomized to patients in which the clinician indicates the test may change management
Consider heterogeneity and local context on test impact	Evaluate center-level effects, consider mixed-methods approaches	RCT ADEQUATE trial: RVP versus SOC demonstrated a 10% decrease in antibiotic prescribing overall, but when stratified by center, significant heterogeneity in effects seen
Utilize observational study design methods and the Target Trial Framework	Design the observational study to mimic an RCT Help avoid common pitfalls with many biases (eg, immortal time bias)	RCT-DUPLICATE study: Demonstrated that this approach yields the same statistical significance in 75% of studies and the same estimate in 66% of studies
Include crude and adjusted estimates	Rather than using statistical methods for causal model selection, use approaches such as directional acyclic graphs (DAGs) and clinical judgement	Stepwise selection and overfitted models may lead to spurious associations and biased regression estimates
Use alternative approaches to analysis	Do not assess an outcome while conditioning on a future event Address immortal time bias Use intention-to-treat or per-protocol analysis, as appropriate	Conditioning on future events: Evaluating the efficacy of a diagnostic among only influenza-positive patients. Immortal time bias: selecting patients who received the result of their test while in the ED versus all patients in the SOC arm for an ED length of stay measurement.

Abbreviations: ED, emergency department; PICOTS, population, intervention, comparison, outcome, timing, and clinical setting; SOC, standard of care; RCT, randomized controlled trial; RVP, respiratory viral panel.

Randomized controlled trials (RCTs) evaluating diagnostic interventions are inherently more complex in design and interpretation than traditional therapeutic trials. Despite clear evidence—from clinical practice and the literature—that local factors such as prescribing norms, guidelines, epidemiology, and provider attitudes strongly influence a test's impact, RCT conclusions often overlook these nuances [15]. Instead, they are often viewed as definitive statements on the generalizable use of RDTs. This disparity underscores a fundamental misunderstanding of diagnostic versus therapeutic research, prompting leading diagnostic methodologists to describe diagnostic RCTs as a “sheep in a wolf's clothing” [16]. Crucially, an RCT can only suggest that an IVD can improve care; it cannot definitively show that it will or will not do so under every clinical practice. The recent multicenter ADEQUATE trial comparing respiratory viral pathogen (RVP) testing with standard of care (SOC) in emergency departments (EDs) exemplifies this principle: although antibiotic prescribing decreased overall by about 10% (*P* < .05), individual centers exhibited highly heterogeneous effects—ranging from substantial reductions to null effects and even increases, all under the same protocol and PICOTS framework (Table 1) [17]. This variation illustrates how essential it is to measure center-level factors influencing impact, yet doing so typically requires resource-intensive multicenter, mixed-methods designs. However, these approaches have been shown to be possible with IVDs in large multicenter observational studies [18]. Research networks could further facilitate future endeavors in this area with RCTs or observational designs. However, given that diagnostic RCTs often cost upwards of a million dollars for single-center studies and nearly half of them fail to complete enrollment, questions remain about the utility of such design and its associated financial expenditures in research [19]. Given these challenges, well-designed observational studies present an appealing, more cost-effective alternative for assessing real-world impact.

While more efficient in resource requirements, future observational IVD studies will require modern epidemiological principles. Current systematic reviews report that approaches to RDT study design usually have moderate to substantial potential for bias and, thus, represent a low quality of evidence [20–23]. To ameliorate these biases, the application of the “target trial framework,” or designing an observational study to emulate RCT data measurement and analysis, facilitates the avoidance of common pitfalls with many biases (eg, immortal time bias) (Supplementary Figure 1). Moreover, as proof of concept, this approach has yielded the same statistical significance in 75% of RCTs and the same effect estimate in 66% of studies in the RCT-DUPLICATE Initiative of 32 RCTs—an initiative started under the 21st Century Cures Act to inform when observational studies could inform regulatory decisions [24].

**Figure 2.**
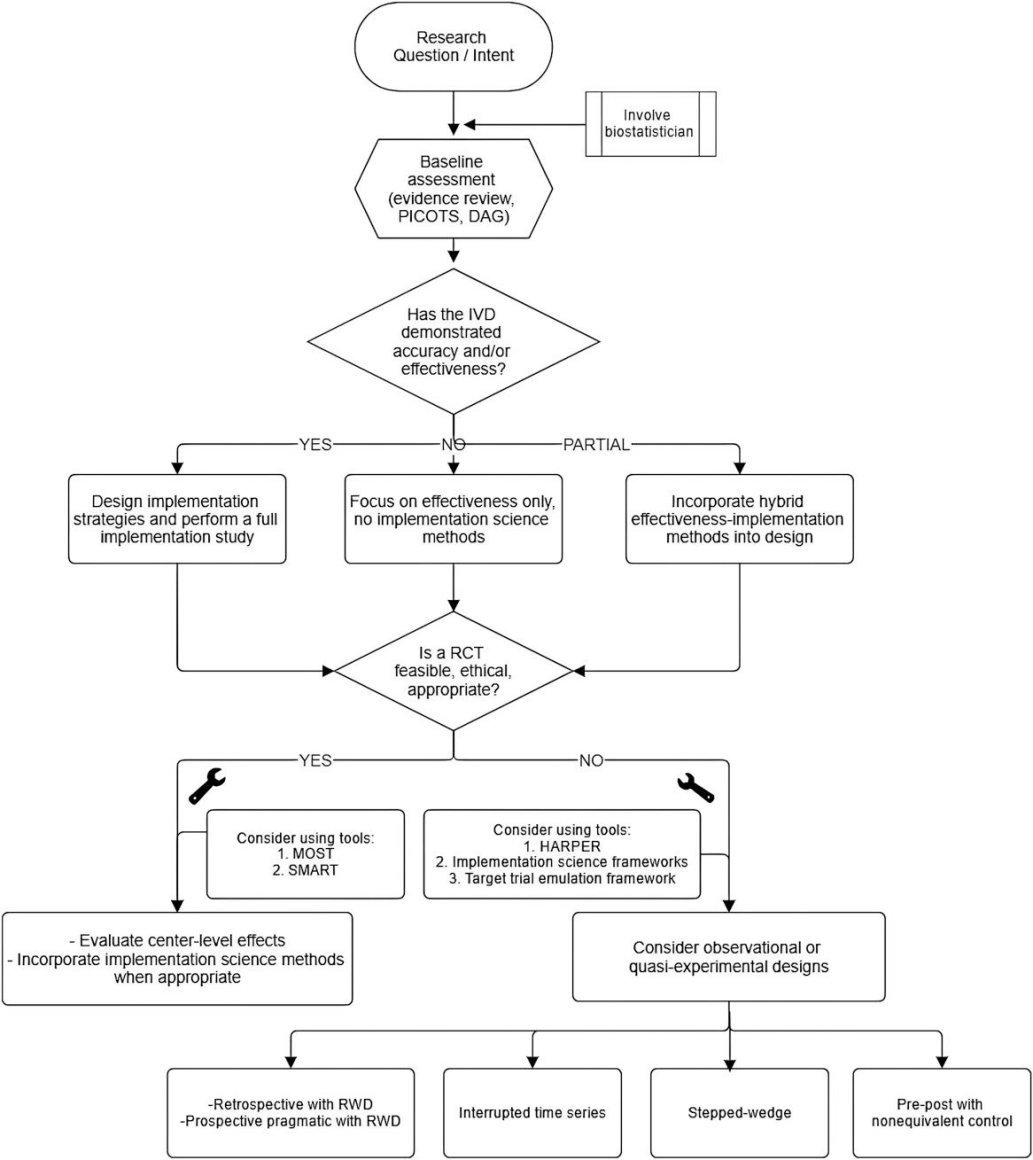
Decision schematic for designing IVD studies. Abbreviations: PICOTS, population, intervention, comparison, outcome, timeframe, setting; DAG, directed acyclic graph; IVD, *in vitro* diagnostic test; RCT, randomized controlled trial; MOST, multiphase optimization strategy trial; SMART, sequential multiple arm randomization trial; HARPER, HARmonized Protocol Template to Enhance Reproducibility.

Opportunities to enhance study design to reduce bias and improve the quality of evidence include providing not only basic estimates of effect but also confounding-adjusted estimates. Such adjusted estimates are typically missing in almost all observational studies of RDTs [20–23]. Moreover, when applied in the more general ID literature, adjustment models often neither specify their goal (ie, description, prediction, or causation), nor follow established recommendations for model development. This is important for the model design approach and study interpretation, so much so that the International Society for Pharmacoepidemiology (ISPE), the Strengthening the Reporting of Observational Studies in Epidemiology (STROBE) Statement, and leading methodological experts in the field, all recommend not using statistical methods (eg, stepwise selection for a prediction model) for causal model covariate selection but instead using approaches like directional acyclic graphs (DAGs) for delineating causal relationships (Figure 2) and clinical judgment to determine causal model covariates for adjustment [25–29].

Improving study design quality leads to decreasing potential bias and increased quality of evidence for informing clinical practice and guidelines. Additionally, these thoughtful approaches to embracing causal frameworks helps avoid adjusting for noncausal variables, which are known to bias estimates. For instance, hemodialysis is usually a confounder—causally associated with both the exposure and outcome—for antibiotic studies but would be an exclusion criterion in a study of procalcitonin. Similarly, penicillin allergy is a confounder for antibiotic studies but not for IVD studies. These examples delineate where misspecifying appropriate confounders for adjustment can lead to biased estimates of effect. Finally, observational studies are still limited akin to RCTs in answering “can it work” for a setting at a point in time rather than “does it work” given the changes inherent with diagnostic efficacy due to center-level effects of local practices (eg, business hours only coverage of RDT), availability of new therapies to improve outcomes with RDT (eg, Paxlovid, ceftazidime–avibactam), learning of RDT over time (eg, clinician will often ignore results of a test when learning to trust the test), and many other factors [30–32].

Finally, for both RCTs and observational studies, there are opportunities for more informative estimates of effect through alternative approaches to analyses. Often in the literature, studies report for a subset of test result patients, such as influenza-positive patients [21]. While these data are informative in elucidating mechanisms of clinical benefit of the test, they are conditioning on future unknowable information (ie, the result of a test) and, thus, are both not an intervention that can be deployed and unable to be appropriately quantified for cost-effectiveness and implementation decision making. Moreover, conditioning on future events may lead to immortal time bias such as selecting patients who received the result of their test while in the ED versus all patients in the SOC arm for an ED length of stay measurement, again highlighting the importance of rigorous modern epidemiological principles and expertise in study design [33]. In contrast to Dr Boten's suggestion that the inclusion criteria require a healthcare provider to acknowledge the test could influence their decision making, studies may alternatively use intention-to-treat (ITT) (ie, the process of analyzing study subject data based on their initial exposure or treatment assignment regardless of not receiving the intervention) as the primary analysis and per-protocol (ie, analysis only of study subjects who adhered strictly to the protocol) as the secondary analysis. This approach captures both a test's general, pragmatic impact and its more explanatory effects when results are employed. Similarly, modified ITT may help to avoid diluting the impact of the intervention when measured among noneligible patients for an outcome (eg, excluding coagulase-negative *Staphylococcus* [CoNS]) contaminants when measuring the mortality impact of RDT in bloodstream infections). Such analysis approaches beyond ITT have been performed historically for procalcitonin trials and have an opportunity to yield explanatory results of the impact of a test under ideal conditions. Recently, intention-to-diagnose has been proposed as a principle that is analogous to ITT used in clinical trials, wherein all enrolled and tested patients remain included in statistical analysis to ensure a more generalizable and less biased evaluation of IVDs performance [34]. The intention-to-diagnose principle includes the consideration for results that either fall into a diagnostic gray zone or are invalid due to application issues or scientific limitations of the IVD.

## NOVEL METHODS FOR STUDYING THE CLINICAL IMPACT OF DIAGNOSTIC TESTS

When a new IVD is approved, the available literature to help clinicians assess their clinical impact on the diagnosis and management of infections is largely limited to *in vitro* studies comparing the new test to a prespecified gold standard [35]. Considerations regarding clinical decision making, antimicrobial resistance (AMR), and antimicrobial and diagnostic stewardship, goals should be assessed, while IVDs are being developed [36–38]. Frequently, however, there is little beyond comparison of sensitivity and specificity of on-panel targets to help clinicians determine which test may best suit their health system or patient population.

The Antibacterial Resistance Leadership Group (ARLG) is a national, federally funded, initiative whose mission is to design and execute clinical trials that enhance the prevention, diagnosis, and treatment of antimicrobial-resistant infections [39–41]. As such, the ARLG has developed novel methodological and statistical approaches to assist researchers in advancing optimal treatment of antibiotic-resistant infections, methods for prevention of AMR, streamlining comparative diagnostic research processes, and providing clinically relevant endpoints for clinicians. This section will review the ARLG-developed Benefit–Risk Evaluation of Diagnostics: A Framework (BED-FRAME), Desirability of Outcome Ranking Management of Antimicrobial Therapy (DOOR-MAT), and Desirability of Outcome Ranking (DOOR), with a comparison in Table 2. Each framework is further described below with examples of applications to IVDs.

**Table 2.** Comparison of ARLG-Developed Methodologies That Can Be Applied to Diagnostic Studies

	BED-FRAME	DOOR-MAT	DOOR
Full name	Benefit–Risk Evaluation of Diagnostics: A Framework (BED-FRAME)	Desirability of Outcome Ranking Management of Antimicrobial Therapy (DOOR-MAT)	Desirability of Outcome Ranking (DOOR)
Primary objective	To guide the assessment of the trade-offs between multiple IVDs and their potential impact on clinical decision making.	To optimize the management of antimicrobial therapy decisions based on organism resistance profiles.	To rank and evaluate the desirability of outcomes between treatment groups.
Methodology/data	Five-step approach. Uses a benefit–risk framework to evaluate IVDs and inform clinical decisions. Prevalence of organisms and AMR data necessary. Relative clinical importance of missed identification/resistance needs to be determined through survey of clinicians.	Six step approach. Extends the ordinal framework design to incorporate matrix-based management of antimicrobial therapy relative to phenotypic susceptibility profiles. Uses data on antimicrobial therapy decisions, AMR profiles, and partial credit scoring analysis for quantitative comparisons.	Employs ordinal outcome ranking to determine the most effective therapy while balancing benefits and risks. Pairwise comparisons indicate the likelihood that a randomly assigned patient in one group will experience better (or worse) outcomes than a patient in another group.
Application area	Direct evaluation of IVDs in the context of AMR.	Indirect evaluation of IVDs through ranking of antimicrobial therapy decisions, specifically with respect to phenotypic antimicrobial resistance.	Evaluation and ranking of clinical outcomes when comparing two different treatment strategies, ideally in a RCT.
Implementation	Requires a structured approach to assess diagnostics and their potential impact on therapy, includes direct comparisons, data abstraction, and survey development.	Focused on ranking and managing the desirability of outcomes in antimicrobial therapy. Includes indirect comparison through downstream decisions.	Focuses on determining the desirability of overall patient outcomes between two interventions in RCTs. Includes indirect comparison through clinical outcomes.
Strengths	Helps clinicians evaluate whether IVDs are beneficial in the context of treatment options specific to their patient populations while incorporating diagnostic stewardship principles.	More granular assessment of complex therapy decisions, allowing for adjustments based on institutional resistance and preferences in antimicrobial therapies (incorporates AMS principles).	Uses real-world clinical outcomes data. Simplified ordinal approach allows for patient-based outcomes analysis. Can still analyze individual outcome components.
Weaknesses	Does not directly address therapy decisions or clinical outcomes. Challenging to determine relative clinical importance. Statistical comparisons may be more challenging for clinicians to interpret.	Can oversimplify antimicrobial therapy decisions. No standardized approach to spectrum of activity determination or partial credit scoring. May be logistically difficult to compare diagnostics outside theoretical comparisons.	Few disease states have standardized DOORs. The heterogeneity in what is included in each rank level can decrease generalizability Identifying and defining clear, mutually exclusive outcomes can be difficult. DOOR may oversimplify the decision-making process by reducing outcomes to a simple ranking, particularly with different types of outcomes. Application in non-RCTs (retrospective cohort studies) is common but can introduce bias.

Abbreviations: AMR, antimicrobial resistance; AMS, antimicrobial stewardship; ARLG, Antibacterial Resistance Leadership Group; IVD, *in vitro* diagnostic test; RCT, randomized controlled trial.

### Comparing *In Vitro* Diagnostics Use the Benefit–Risk Evaluation of Diagnostics: A Framework

The ARLG BED-FRAME was developed to assist in choosing between IVDs while considering the impact of local AMR prevalence and potential downstream clinical impact [42]. This framework was developed to be supplementary to traditional IVD evaluations. The BED-FRAME consists of five steps focusing on visualization of the test risks and benefits as localized to a specific population of interest. The ARLG provides examples of the application of components of BED-FRAME to compare IVDs that detect carbapenem-resistant Enterobacterales, *Acinetobacter baumannii*, and *P. aeruginosa* through the PRIMER studies [43–45]. Pragmatic analysis using the intention-to-diagnose principle is exemplified with BED-FRAME [34]. In particular, BED-FRAME could assist diagnostic stewardship and AMS programs in determining which IVDs would be best for their institutions prior to onboarding by considering patterns of resistance and how missed detection of that resistance could impact optimal patient management.

Published real-world examples of BED-FRAME outside the ARLG are limited. Smith et al [46] used a modified version of BED-FRAME to compare three RDT platforms for blood culture organism identification and resistance detection. This single-center study collected 301 monomicrobial-positive blood culture samples from unique patients. Pathogen prevalence was determined through local clinical microbiology data, and relative clinical importance and preferred diagnostic attributes were determined by surveying clinicians and AMS team members. The sensitivity of all three RDTs compared with culture results was over 88%. As determined by the AMS team, the most important diagnostic attributes were accurate pathogen identification (77.8%), identifying resistance determinants (16.7%), and time-to-results (5.6%). Following the steps of BED-FRAME, health systems can compare, through direct or theoretical application, different IVDs and balance stewardship priorities with laboratory workflow.

### Assessing Antibiotic Use Through the Desirability of Outcome Ranking Management of Antimicrobial Therapy Framework

Many scoring tools have been developed to quantify antimicrobial spectrum of activity for intra- and interfacility benchmarking beyond traditional antimicrobial days of therapy [47–49]. Optimal or desirable therapy is generally considered the narrowest spectrum with *in vitro* activity, but this simplified definition is often difficult to apply pragmatically [50]. In particular, these scoring tools do not allow for consideration of AMR profiles and solely focus on spectrum [40, 51, 52]. The DOOR-MAT is a novel framework to assess antimicrobial therapy decisions considering both antimicrobial spectrum of activity and the organism AMR patterns. The DOOR-MAT cross-references the prescribed antimicrobial to the final organism resistance profile and applies a score of 0 (inactive) to 100 (active and most narrow). It is unique in its structure and ability to aid clinicians in comparing therapy decisions in a quantifiable manner given patient-specific characteristics and diagnostic or AMS goals. Studies have published examples of DOOR-MAT frameworks that can be applied for comparisons specific to Enterobacterales [51–53]. These can be used as a starting point and tailored to individual health system formularies or prescribing patterns.

Two studies have leveraged DOOR-MAT to compare theoretical antimicrobial therapy decisions based on different blood RDT panels [54, 55]. These studies adapted the DOOR-MAT developed by Wilson et al to a series of clinical blood culture isolates tested with different RDT panels. The results were compared to traditional culture and automated antimicrobial susceptibility testing. For each patient, RDT results were presented with case vignettes addressing clinical considerations such as age, infection severity, infection source, and history of AMR. Based on the results from the different panels, two clinicians made theoretical antimicrobial therapy decisions. With a high rate of agreement between prescribing clinicians, these decisions were then referenced against the DOOR-MAT framework considering the final phenotypic resistance profiles of the detected organisms. The studies highlighted how traditional metrics such as positive percent agreement (PPA) do not accurately reflect the impact of different panels on antimicrobial therapy decisions. For instance, while two panels showed similar PPAs {98% (95% confidence interval [CI] 94.3% to 99.6%) vs 98.6% (95% CI 95.1% to 99.8%)}, their mean DOOR-MAT scores differed significantly {a score of 91.9 (standard deviation [SD] ± 23.1) vs 86.8 (SD ± 28.5)} [52]. A second study compared two panels, one of which had expanded organism and resistance detection [51], and found that even though both had high sensitivity, the expanded panel had a much higher DOOR-MAT score (59.9 [SD ± 33.7] vs 89.7 [SD ± 24.7]), underscoring its role in in optimizing antimicrobial therapy.

### Determining Patient Clinical Outcomes With the Desirability of Outcomes Ranking Framework

Both BED-FRAME and DOOR-MAT allow for direct or indirect comparison of IVDs, respectively, by considering local AMR profiles and incorporating diagnostic stewardship and AMS principles. They do not, however, allow for assessment of patient clinical outcomes. This is where the DOOR is useful [56]. The DOOR allows for holistic patient-centric comparisons of clinical outcomes by combining multiple endpoints (ie, efficacy and adverse events) in a ranked ordinal fashion. Patient outcomes are mutually ranked from range the best outcome (alive with no adverse events) to the worst outcome (mortality). After each patient is assigned a DOOR level, distribution of levels between groups is compared as is the overall probability of a better outcome.

The DOOR is often combined with a continuous variable to assist as a tiebreaker among the pairwise comparisons of DOOR levels. The most common tiebreaker is Response Adjusted for Duration of Antibiotic Risk, which evaluates antibiotic duration under the AMS principle that shorter is better [57]. When patients are assigned the same DOOR level, shorter duration is used to determine which patient has an overall better outcome. Other continuous variables that could potentially be leveraged include health-related quality-of-life measures or DOOR-MAT scoring to consider the spectrum of antimicrobial activity [53, 58].

The DOOR framework has mainly been applied to post hoc evaluations of RCTs comparing novel antimicrobials to SOC for infections such complicated intraabdominal infections or hospital-acquired pneumonia, multidrug-resistant infections (CRACKLE-2), as well as for comparing antimicrobial therapy regimens such as combination beta-lactam/vancomycin (CAMERA-2) and vancomycin area-under-the-curve (PROVIDE) [59–65]. One small quasiexperimental study used DOOR to compare patient outcomes pre- versus postimplementation of blood culture RDTs [66]. Researchers used a 3-level DOOR that was based on the ability to appropriately escalate or deescalate therapy according to the spectrum-of-activity scoring system. The authors reported a 58% (95% CI 54%–62%) overall probability of better outcomes in the post-RDT group compared with the pre-RDT group. Another small study compared the role beta-D glucan testing in optimizing therapy for candidemia in critically ill patients [67]. Numerous opportunities exist for large-scale high-quality IVD studies that incorporate DOOR as a primary or secondary endpoint.

The DOOR methodology has limitations, however, primarily due to its heterogeneous development and application [68]. This includes variation in the number of levels and endpoints considered within each level, as well as the study designs using DOOR. Some studies have developed DOORs with limited rigor, while others rely on consensus methods, such as modified Delphi techniques [66, 69]. Instead of developing DOORs for each study ad hoc, the ARLG has consensus-based DOORs for specific infections, such as *Staphylococcus aureus* BSI and HAP/VAP, which can be modified to enhance study validity [62, 70]. An additional potential limitation to the DOOR, however, is that it has the potential to obscure clinical outcomes by including multiple outcomes of different severity into a single level. Additionally, DOOR was originally developed and applied to results from RCTs, decreasing risk of bias in treatment or group assignments. Many published DOOR studies, however, have applied the method to retrospective cohort data, with or without additional statistical methods such as inverse probability of treatment weighting to help reduce risk of selection bias. Overall, more work is needed to standardize the methodological approach to allow investigators to develop disease-specific scores beyond what has been studied in a manner consistent with ARLG's previous tools.

### Introduction to Hybrid Effectiveness–Implementation Study Framework

Hybrid effectiveness–implementation studies can improve timely use of novel IVDs while also considering key diagnostic stewardship metrics and outcomes. Proposed by Curran et al [71] in 2012, these study designs aim to bridge the gap between evidence-based interventions and practical application. Hybrid studies simultaneously assess the intervention effectiveness and implementation strategy, allowing researchers to evaluate not only how well an intervention works in real-world settings but also how it is integrated and adopted by the target population [71, 72]. There are three different types of hybrid designs, each balancing effectiveness and implementation with differing degrees of emphasis (Table 3). Initially intended for clinical patient-level RCTs, their scope has expanded to include cluster-randomized, stepped wedge and other quasiexperimental studies [73]. Curran et al offer guidance on selecting the right hybrid design that fits with specific types of research questions, considering factors such as existing evidence for the intervention, the extent of adoption necessary, and known barriers and facilitators to implementation. Regardless of the hybrid type and study design selected, leveraging hybrid effectiveness–implementation studies aligns perfectly with the core principles of diagnostic stewardship, which aims to optimize real-world application of diagnostic tools while ensuring they improve patient care [37].

**Table 3.** Comparison and Diagnostic Test Examples of Hybrid Effectiveness–Implementation Studies

Hybrid Type	Primary Focus	Secondary Focus	Example
Type 1	Effectiveness of an intervention	Preliminary implementation data	A patient-level RCT testing a novel blood RDT with phenotypic AST for FDA approval while documenting provider adoption barriers.
Type 2	Both effectiveness and implementation strategies	N/A	Cluster-randomized study of an available RDT for BSI in acute care settings while comparing different AMS strategies to adoption (eg, implementing a new RDT–treatment algorithm, educational tools).
Type 3	Implementation strategy	Effectiveness of an intervention	Comparing approved IVDs with paired strategies (eg, electronic health record [EHR]-based interventions) to reduce unnecessary *C. difficile* testing while assessing the impact on patient management.

Abbreviations: AST, automated susceptibility testing; FDA, Food and Drug Administration; RCT, randomized controlled trial; RDT, rapid diagnostic test.

Since their inception, hundreds of hybrid effectiveness–implementation studies of healthcare interventions have been conducted [73]. These interventions include expanding age-friendly healthcare in VA medical centers, implementing cognitive behavioral therapy, evaluating chronic pain, and more [74–77]. Few have focused on ID, and even fewer on implementing novel IVDs [78–80]. Table 3 provides brief examples of potential applications of hybrid study designs for IVDs. Another example could be a hybrid type 2 trial, using a quasiexperimental design assessing the impact and implementation of a point-of-care (POC) respiratory RDT in children. The coprimary outcomes would be the effect of the POC RDT on effectiveness (ie, decreased antimicrobial use), implementation (ie, acceptability, adoption, feasibility), service (ie, efficiency, equity, timeliness), and client (ie, satisfaction) outcomes. By collecting effectiveness metrics and implementation data concurrently, the hybrid design maximizes research efficiency, as they reduce the need for separate studies focused solely on either aspect. The conventional sequential research approach—from efficacy to effectiveness to implementation—can be time-consuming, delaying the practical application of an intervention. This integrated approach provides a more holistic understanding of an intervention's impact, offering insights into both its clinical outcomes and the practical challenges of its adoption, scalability, and sustainability. This is particularly valuable for interventions where the success of an intervention is often influenced by factors such as provider behavior and organizational context, which is often the case with novel diagnostic tests.

## BROADER IMPACT OF EVIDENCE GENERATION FOR DIAGNOSTICS

The Fryback and Thornbury (FT) hierarchical framework of efficacy was originally created to appraise diagnostic imaging literature but has since been applied to other IVDs [81]. It consists of six levels, focusing on different aspects of diagnostic efficiency. Levels 1 and 2 focus on technical efficacy and diagnostic accuracy, metrics commonly evaluated during clinical trials and the FDA clearance process. Levels 3 through 6 encompass diagnostic thinking efficacy, therapeutic efficacy, patient outcome efficacy, and societal efficacy, respectively. Fryback and Thornbury emphasize that while each level is necessary, none is sufficient alone. For example, level 3 examines whether diagnostic information influences provider thinking, impacting level 4 (changes to patient management). This connection also affects levels 5 and 6, which evaluate patient outcomes and broader societal implications, respectively.

Major payers, such as the Centers for Medicare and Medicaid Services, utilize this framework, among others, to determine what is necessary and reasonable for reimbursement [82]. *In vitro* diagnostic test clinical trials (ie, premarket launch) often produce substantial data for FDA submissions that focus on technical and diagnostic efficacy (ie, levels 1 and 2 of the FT framework), which is crucial for safety and efficacy demonstration. However, these data may not suffice for payers’ coverage decisions. A review of Health Technology Assessment (HTA) methodologies demonstrated that while approaches may vary, there is a consensus that accuracy alone cannot establish clinical effectiveness [83]. The way evidence is generated is crucial for test adoption and reimbursement; a PICOTS framework, highlighted earlier in this paper, should always be utilized, and the role of a new IVD in the current care pathway should be demonstrated whenever possible.

Direct evidence, which involves comparing clinical outcomes following different test strategies within the same primary study, typically in the form of RCTs that randomize by test method, is preferred by payers and HTA agencies [83]. This is especially true for novel molecular IVDs, where providers often depend on indirect evidence, while payers rely on clinical guidelines and results from RCTs [84]. However, direct diagnostic evidence that includes treatment pathways (eg, test-plus-treatment RCTs) is rare and has considerable limitations. Reviews of test–treatment RCTs indicate that they are more susceptible to methodological challenges than RCTs for drugs [83]. Furthermore, the controlled nature and enrollment requirements of such trials can make them infeasible [85]. Conducting high-quality RCTs for IVDs is challenging due to factors like blinding, learning effects (eg, the user learning curve), selecting appropriate comparators, and changes in IVD performance over time.

### Real-world Data and Real-world Evidence

To supplement RCT data and support clinical guideline development and payer coverage, real-world data (RWD) has emerged as a viable option to generate real-world evidence (RWE) [85, 86]. The 2024–2025 International Society for Pharmacoeconomics and Outcomes Research report lists RWE as the top trend in health research. Real-world evidence, which uses data from routine healthcare delivery (eg, electronic health records, insurance claims databases, data from wearable devices), offers a more realistic view of diagnostic interventions and outcomes and has gained support from various agencies, including the FDA, European Medicines Agency, National Institute for Health and Care Excellence, and Canada's Drug and Health Technology Agency. A 2018 Institute for Clinical and Economic Review (ICER) publication on RWE for coverage decisions highlights that the healthcare community often operates under outdated evidence hierarchies that regard RCTs as the single gold standard for evidence generation and healthcare decision making [87]. These hierarchies were not designed for a world driven by big data, where RWE can supplement RCTs and enhance our understanding of the effectiveness of interventions across various populations. While RWE is increasingly utilized in IVD regulatory decision making, hesitations remain in the postlaunch interventional and clinical outcomes space due to potential biases in nonrandomized studies and perceived flaws in study design [82, 88]. Ultimately, a lack of confidence in observational studies has slowed uptake of RWE into policy [89]. Therefore, studies must be rigorously designed to minimize bias. This rigor is crucial, as robust RWE plays a significant role in multiple healthcare decisions, including HTAs, payer decisions, clinical guidelines, and health policy [87, 90].

Real-world evidence may be generated using various study designs and with data acquired through routine health practice or real-world settings. The most common nonrandomized study types using RWE to assess comparative effects of IVDs and pharmaceuticals are observational cohort studies and single-arm trials with a real-world external control. These methodologies are often employed when full RCTs are uncommon and help address qualitative considerations, such as patient experiences with an IVD or intervention [91]. Additionally, pragmatic clinical trials, or trials that are randomized and contain varying levels of RWD elements, are recommended as strong alternative approaches [89]. Real-world evidence is used throughout an IVD product life cycle, including leveraging data to identify future innovation, clinical trials for FDA submission, effectiveness studies, and label expansion [92]. The FDA provides a summary document detailing 90 examples of when RWD was used for regulatory decision making in the premarket and postmarket processes for IVDs, including label expansions, surveillance, and validation [88].

The potential for using RWD to generate RWE after the launch of an IVD is significant. This includes assessing test effects in routine settings, conducting head-to-head comparisons, evaluating populations often excluded from RCTs, and exploring intervention effect variability [91]. An example of the use of RWD to assess the impact of IVDs in the postlaunch period is seen in a 2023 study by Moon et al [93] which utilized hospital discharge data from the PINC AI Healthcare Database to understand the demographic and clinical characteristics, epidemiology, healthcare resource utilization, and cost among adult outpatients with acute gastroenteritis visiting US health systems. Furthermore, a recent publication by Fitzke et al [94] highlights study examples where RWD was used to answer study questions in ID, preventative medicine, and HTAs.

To effectively adopt IVDs, healthcare decision makers require evidence that is reliable, relevant, and robust, addressing clinical benefits and cost-effectiveness while providing recommendations for specific subpopulations [95]. Each study should include a clear research question and relevant patient outcomes that reflect how patients feel, function, or how long they live. The Core Outcome Measure in Effectiveness Trials database can be useful for identifying important outcomes [89, 96]. Additionally, researchers should prespecify study plans with tools such as the HARmonized Protocol Template to Enhance Reproducibility (HARPER) and are encouraged to register their protocols before starting the study [89, 97]. It is important to select appropriate data and conduct a systematic search early on to address key gaps. Strategies to reduce bias in sampling should include stratified or purposive sampling. The analytic design should align with the research question and data characteristics, including outcome distribution and sample size. During the study, diagnostic data checks should identify and mitigate potential biases, such as inconsistent variable definitions or missing data. Finally, transparency in reporting methods is crucial, especially when indirect or data-linking evidence methods are used. Involvement of key stakeholders, particularly payers, throughout the study design process is essential for gaining support and access approval [82, 84, 85, 98]. Finally, utilizing methods that support reliable causal inference alongside RWE is vital for building the “totality of evidence” for the IVD [99].

### Where to Start When Designing Clinical Outcomes Studies for *In Vitro* Diagnostic Test

Designing a clinical outcomes study for IVDs can be challenging for general ID clinicians or clinical microbiologists. While engaging in clinical trial design and conducting performance studies are essential for the clearance and use of IVD products, this section will focus on designing clinical impact and utility studies conducted after a product has been launched and is available on the market (Figure 2). The foundation of any study lies in its research question and objectives. A crucial first step is to understand baseline data, which include existing evidence, gaps, patient populations of interest, and relevant epidemiological factors. This information is vital for identifying PICOTS and creating a conceptual model or DAG that clarifies the pathways and processes influencing study design and outcomes.

It is essential to involve a biostatistician as early as possible, especially when defining the study's outcomes, determining the appropriate study power, and calculating the necessary sample size to accurately measure those outcomes. The researcher should consider whether a single outcome or a composite outcome would more appropriately address the research question. In cases of AMR-focused research, employing composite outcome methodology, like the DOOR framework, may effectively convey a more comprehensive clinical narrative. Once the study's power, sample size, and outcomes are established, the researcher should also consider whether implementation science methodologies could enhance the study (Figure 2). Although implementation science is a relatively new field in clinical research, especially in the context of ID IVD studies, it offers valuable insights into how a test may be adopted and the contextual factors impacting clinical outcomes. Understanding that the clinical outcomes linked to IVD test results are influenced by human behavior is key; consequently, accounting for human behavior, psychology, and the local environment is crucial for interpreting these outcomes. A publication by Lane-Fall et al [100] encourages researchers to position themselves within the translational science pipeline while designing their studies, identifying moments when integrating implementation science methodologies might be beneficial.

Researchers should evaluate whether the effectiveness of the IVD has been established in the literature. For IVDs, this typically means demonstrating diagnostic accuracy and some degree of clinical impact. If there are significant data on performance and clinical utility (affirmative) or considerable data on accuracy and safety but incomplete data on clinical effectiveness (partially affirmative), the researcher should collect implementation-related data to improve adoption, access, or contextualize research findings. If there are no supportive data, indicating that the intervention is not yet ready for implementation, the researcher should refrain from incorporating implementation science methods and focus solely on demonstrating effectiveness. In instances where effectiveness is only partially demonstrated, hybrid implementation–effectiveness designs may be appropriate; guidance on designing such trials is available in other resources [71, 72].

Next, regardless of whether implementation science methodologies are used, researchers should assess whether a RCT is reasonable, ethical, and suitable. While RCTs are widely regarded as the gold standard in clinical research, certain scenarios may not favor them as the optimal design for studying the impact of an IVD. If an RCT is deemed the best design choice, researchers should adhere to best practices for RCTs and strive to enhance the generalizability and applicability of the study findings, including integrating implementation science methodologies. Furthermore, for complex interventions with multiple components, such as AMS interventions, researchers should seek to understand the impact of individual components rather than evaluating the intervention through post hoc analyses. In such cases, the Multiphase Optimization Strategy (MOST) can be used to analyze the effects of individual intervention components before conducting the RCT [101]. The MOST involves three phases (eg, screening, refining, and confirming) to identify components most likely to have a significant impact while meeting predefined criteria (eg, cost). Another innovative design, the Sequential Multiple Assignment Randomized Trial (SMART), may be beneficial for evaluating time-varying adaptive interventions, helping researchers understand the influence of the sequence of intervention components and the optimal timing for each component. Both the MOST and SMART are advanced design methods applicable to interventions that involve behavioral elements, as seen with IVDs. Detailed considerations and steps for implementing these methods have been published elsewhere [102].

If a researcher concludes that a RCT is not feasible, ethical, or suitable for addressing the study question, they should consider observational and quasiexperimental designs. It is important to recognize that data from observational RWE can effectively complement or expand on data from RCTs. Observational studies, especially retrospective ones that utilize large datasets (such as claims databases), can be particularly valuable for research focusing on health economics outcomes. Pragmatic trials, which are prospective randomized trials that leverage RWD, can help overcome some limitations of observational studies and improve the generalizability of findings [103]. However, challenges may arise related to feasibility and the balance between internal and external generalizability. Tools such as HARPER are available to guide researchers in designing RWE studies that enhance transparency and facilitate reproducibility. Furthermore, investigators may consider the use of approaches intended to help overcome significant limitations of observational research, such as the target trial emulation framework. One way to ensure that the analyses in observational studies preserve the optimal features of RCTs is to design them so that they emulate a hypothetical RCT [104]. To do this, the investigator should write the study protocol to include key elements such as eligibility criteria, treatment strategies, treatment assignment, the start and end of follow-up, outcomes, and the data analysis plan. Next, observational data can be used to identify eligible individuals and assign them to a treatment strategy according to their data and match the study analysis to what would be done during an RCT apart from adjusting for baseline confounders, which is done to emulate random treatment assignment [104, 105]. Taking this approach encourages investigators to align treatment assignment and follow-up at time zero, which helps avoid significant biases seen in observational research such as immortal time bias, lead time bias, and selection bias [105].

Several robust quasiexperimental designs exist that provide rigor while requiring fewer resources, making them excellent options for studies on clinical impact and implementation science of IVDs. The three most commonly utilized designs are interrupted time series, stepped wedge, and pre–post with a nonequivalent control. These designs are effective for assessing outcomes before and after the implementation of an IVD or related intervention and can incorporate methodologies from implementation science [106]. Advanced quasiexperimental approaches build on basic quasiexperimental methodologies by incorporating techniques that improve causal inference [107]. For example, a pre–post design with a control group and a difference-in-differences analysis addresses underlying time-dependent trends, whereas a simple pre–post design does not [108].

## CONCLUSIONS

Evaluating the clinical impact of IVDs is complex and closely tied to various factors such as clinician behavior, treatment pathways, culture, and learning, requiring innovative approaches to study design and methodological rigor. It is essential to consider and adjust for the local context and to involve key stakeholders throughout all stages of study design and execution, including payors and policymakers when appropriate. Those engaged in clinical outcomes research related to IVDs should strive to advance the field by incorporating these methodologies and demonstrating their effectiveness. A thoughtful and comprehensive approach to studying the impact of IVDs has the potential to enhance patient care, improve public health, and increase access to better diagnostic testing.

## Supplementary Material

ofaf489_Supplementary_Data

## Supplementary Data

Supplementary materials are available at *Open Forum Infectious Diseases* online. Consisting of data provided by the authors to benefit the reader, the posted materials are not copyedited and are the sole responsibility of the authors, so questions or comments should be addressed to the corresponding author.
