# Supplementary material for: Beyond Accuracy: Methodological Advances for Assessing the Clinical Impact of Infectious Disease Diagnostics
Source: Open Forum Infect Dis. 2025 Oct 22;12(Suppl 2):S1391–403. doi: 10.1093/ofid/ofaf489 (PMC12541908; doi:10.1093/ofid/ofaf489)
Supplement: ofaf489_Supplementary_Data [file ofaf489_supplementary_data.docx]

**Supplemental materials**


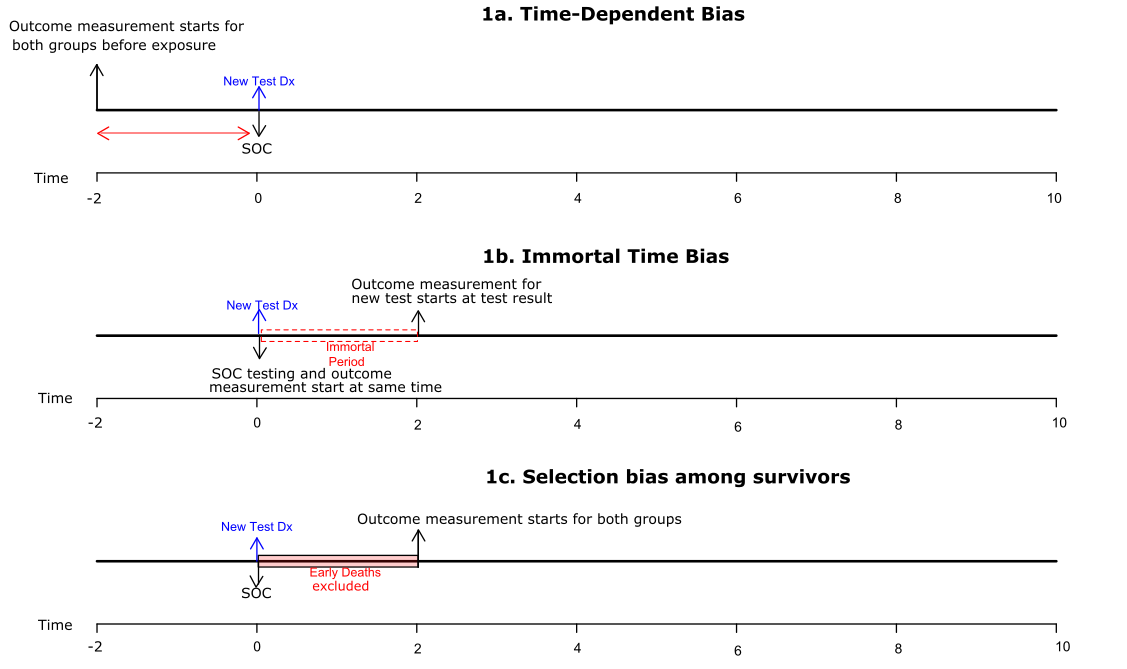


**Supplemental Figure 1.** Three Example Biases in Diagnostic Studies. 1a. Time-dependent bias: Starting outcome measurement starts at the same time for both arms but starting measurement before interventions biases estimate of effect. 1b. Immortal Time Bias: Patients in the new‐test arm must survive (e.g., not discharged) long enough to receive the test result, creating an “immortal” window (e.g., artificially extending emergency department length of stay) and skewing outcomes. 1c. Selection Bias Among Survivors: Excluding early deaths (e.g., within 48 hours) from both groups removes the sickest patients post hoc, potentially obscuring the true benefit for those who might gain most from timely treatment.
